# Author Correction: Murine liver repair via transient activation of regenerative pathways in hepatocytes using lipid nanoparticle-complexed nucleoside-modified mRNA

**DOI:** 10.1038/s41467-021-23322-6

**Published:** 2021-05-10

**Authors:** Fatima Rizvi, Elissa Everton, Anna R. Smith, Hua Liu, Elizabeth Osota, Mitchell Beattie, Ying Tam, Norbert Pardi, Drew Weissman, Valerie Gouon-Evans

**Affiliations:** 1 Center for Regenerative Medicine and the Section of Gastroenterology of Boston University and Boston Medical Center, 670 Albany street, Boston, MA 02118 USA; 2 Acuitas Therapeutics, Vancouver, BC V6T 1Z3 Canada; 3 Department of Medicine, University of Pennsylvania, Philadelphia, PA 19104 USA

**Keywords:** Biological techniques, Biotechnology, Cell biology, Diseases, Gastroenterology

Correction to: *Nature Communications* 10.1038/s41467-021-20903-3, published online 27 January 2021.

In the original version of the published article, the Acknowledgements section was missing a statement to acknowledge funding from an Alpha-1 Foundation Research grant. The correct Acknowledgements paragraph is:

