# Supplementary material for: Murine liver repair via transient activation of regenerative pathways in hepatocytes using lipid nanoparticle-complexed nucleoside-modified mRNA
Source: Nat Commun. 2021 Jan 27;12:613. doi: 10.1038/s41467-021-20903-3 (PMC7840919; doi:10.1038/s41467-021-20903-3)
Supplement: Supplementary file 1 — Supplementary Information [file 41467_2021_20903_MOESM1_ESM.pdf]

SUPPLEMENTARY INFORMATION

Fatima Rizvi [1,*], Elissa Everton [1,*], Anna R. Smith [1], Hua Liu [1], Elizabeth Osota [1], Mitchell Beattie [2], Ying Tam [2], Norbert Pardi [3], Drew Weissman [3], Valerie Gouon-Evans [1*]. Murine liver repair via transient activation of regenerative pathways in hepatocytes using lipid nanoparticle-complexed nucleoside-modified mRNA.

[*] These authors contributed equally

A. Supplementary Figures:

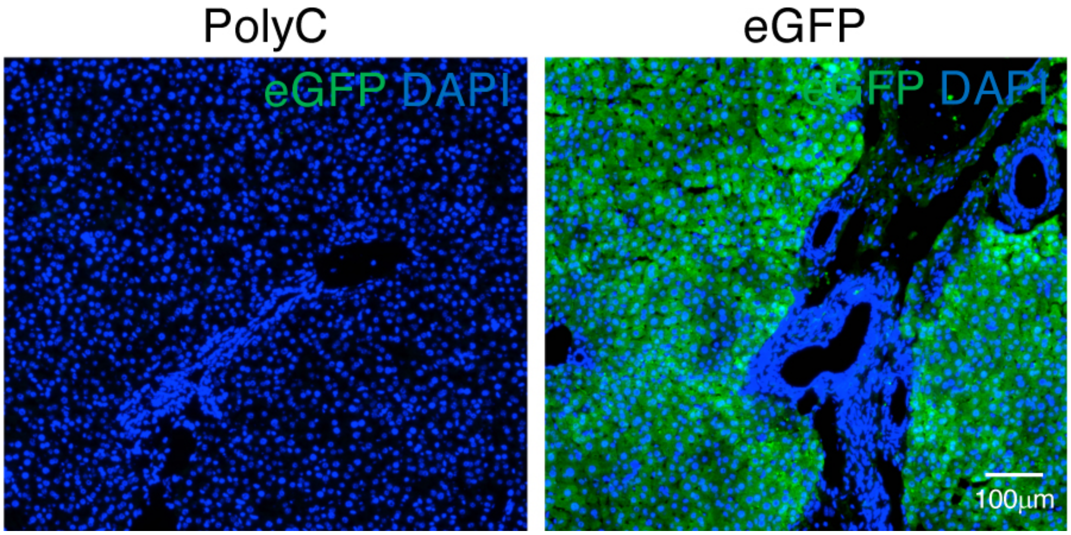

Supplementary Fig. 1 Hepatocytes are the main liver cell type targeted by eGFP mRNA-LNP. GFP immunostaining on liver sections from mice 5 hours after injection with a single dose of Poly(C) RNA-LNP or eGFP mRNA-LNP. Note GFP immunostaining in virtually all hepatocytes. 100X magnification pictures of representative images from 3 mice injected with eGFP-mRNA-LNP and 2 mice injected with Poly(C) RNA-LNP are shown. The scale bar represents 100μm for both 100X magnification images.

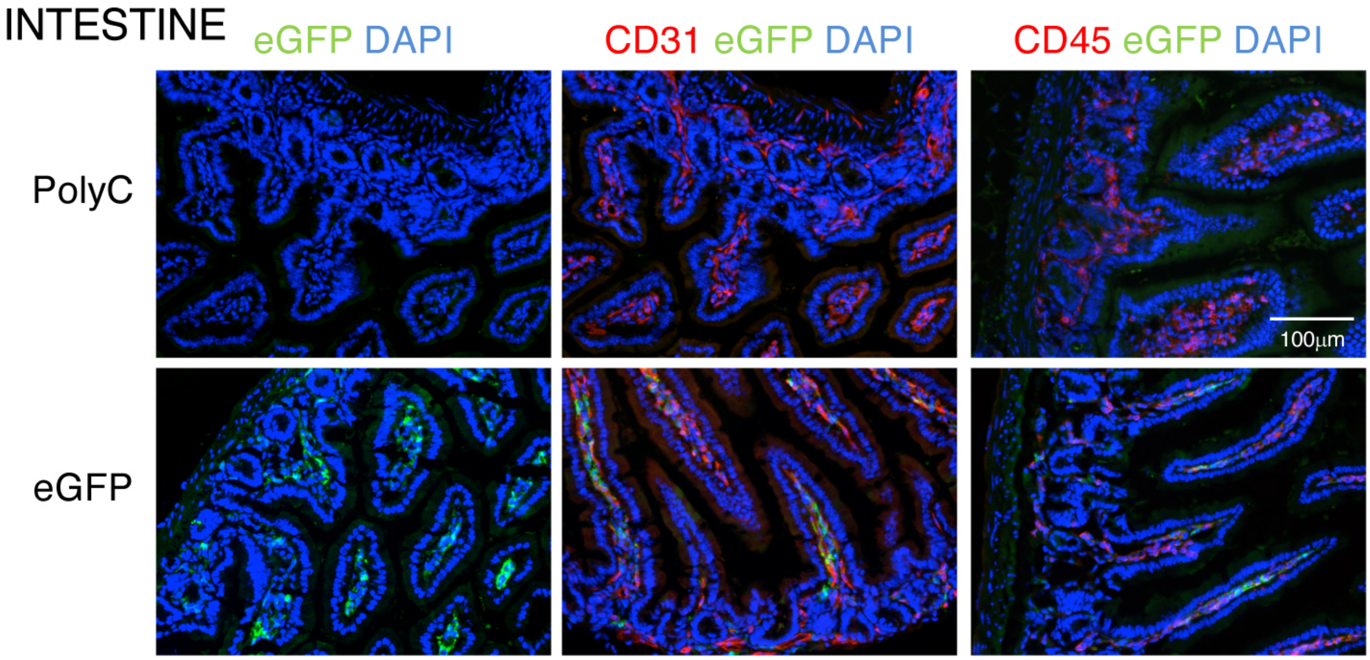

Supplementary Fig. 2 Subpopulations of blood and endothelial cells of the intestine are targeted by eGFP mRNA-LNP. Immunostaining for GFP and co-immunostaining for GFP/CD31 and GFP/CD45 are shown from intestine sections from mice injected with a single dose of Poly(C) RNA-LNP or eGFP mRNA-LNP. In intestine, eGFP+ cells are CD31+ endothelial cells or CD45+ blood cells. 100X magnification pictures of representative images from 3 mice injected with eGFP-mRNA-LNP are shown. The scale bar represents 100μm for all 100X magnification images.

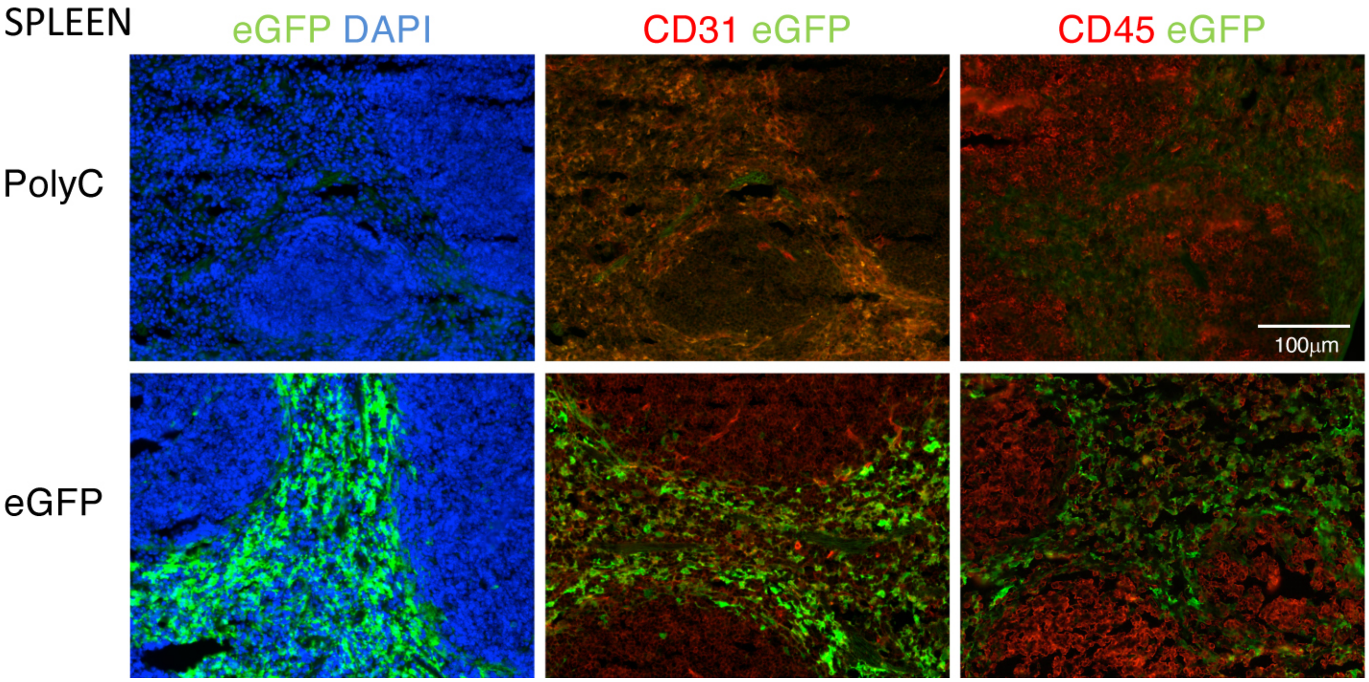

Supplementary Fig. 3 Subpopulations of blood and endothelial cells of the spleen are targeted by eGFP mRNA-LNP. Immunostaining for GFP and co-immunostaining for GFP/CD31 and GFP/CD45 are shown from spleen sections from mice injected with a single dose of Poly(C) RNA-LNP or eGFP mRNA-LNP. In the spleen, eGFP+ cells are CD31+ endothelial cells or CD45+ blood cells. 100X magnification pictures of representative images from 3 mice injected with eGFP-mRNA-LNP are shown. The scale bar represents 100μm for all 100X magnification images.

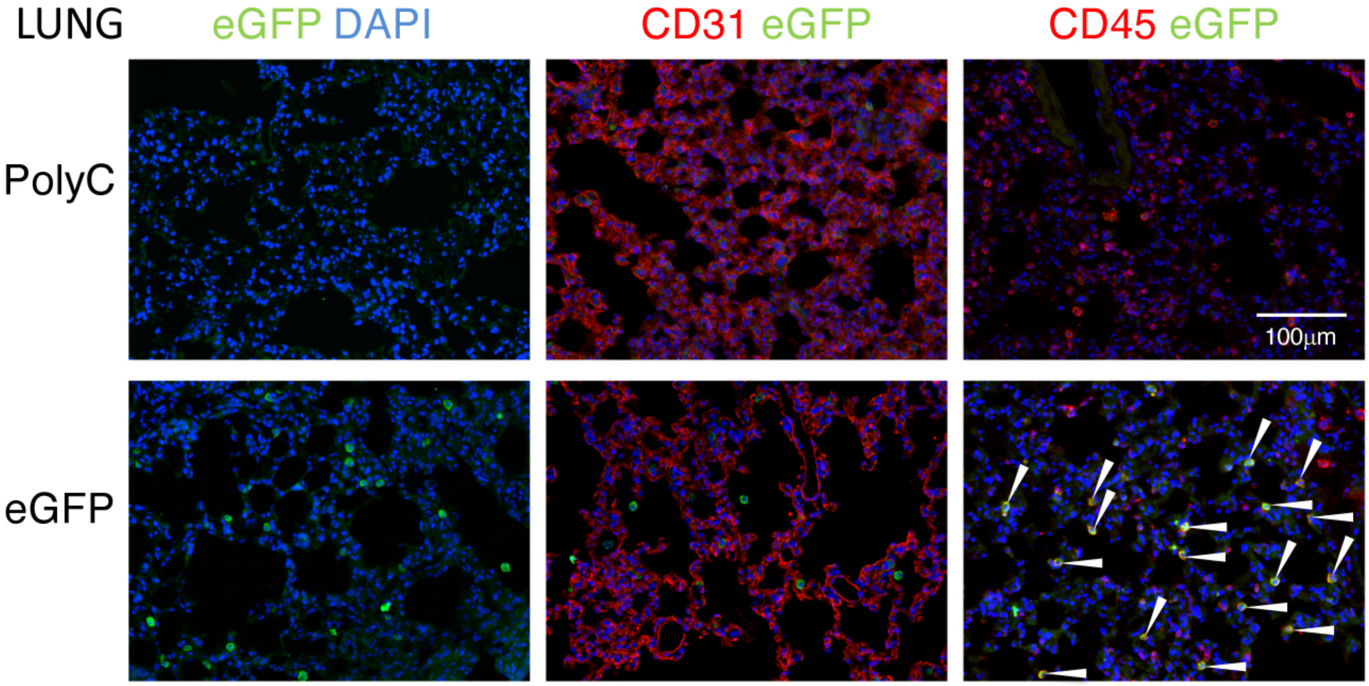

Supplementary Fig. 4 Subpopulations of blood cells, most likely phagocytic cells of the lung, are targeted by eGFP mRNA-LNP. Immunostaining for GFP and co-immunostaining for GFP/CD31 and GFP/CD45 are shown from lung sections from mice injected with a single dose of Poly(C) RNA-LNP or eGFP mRNA-LNP. White arrowheads represent co-stained GFP^{+} $CD45^{+}$ cells. In the lung, eGFP+ cells are mainly CD45+ blood cells. 100X magnification pictures of representative images from 3 mice injected with eGFP-mRNA-LNP are shown. The scale bar represents 100µm for all 100X magnification images.

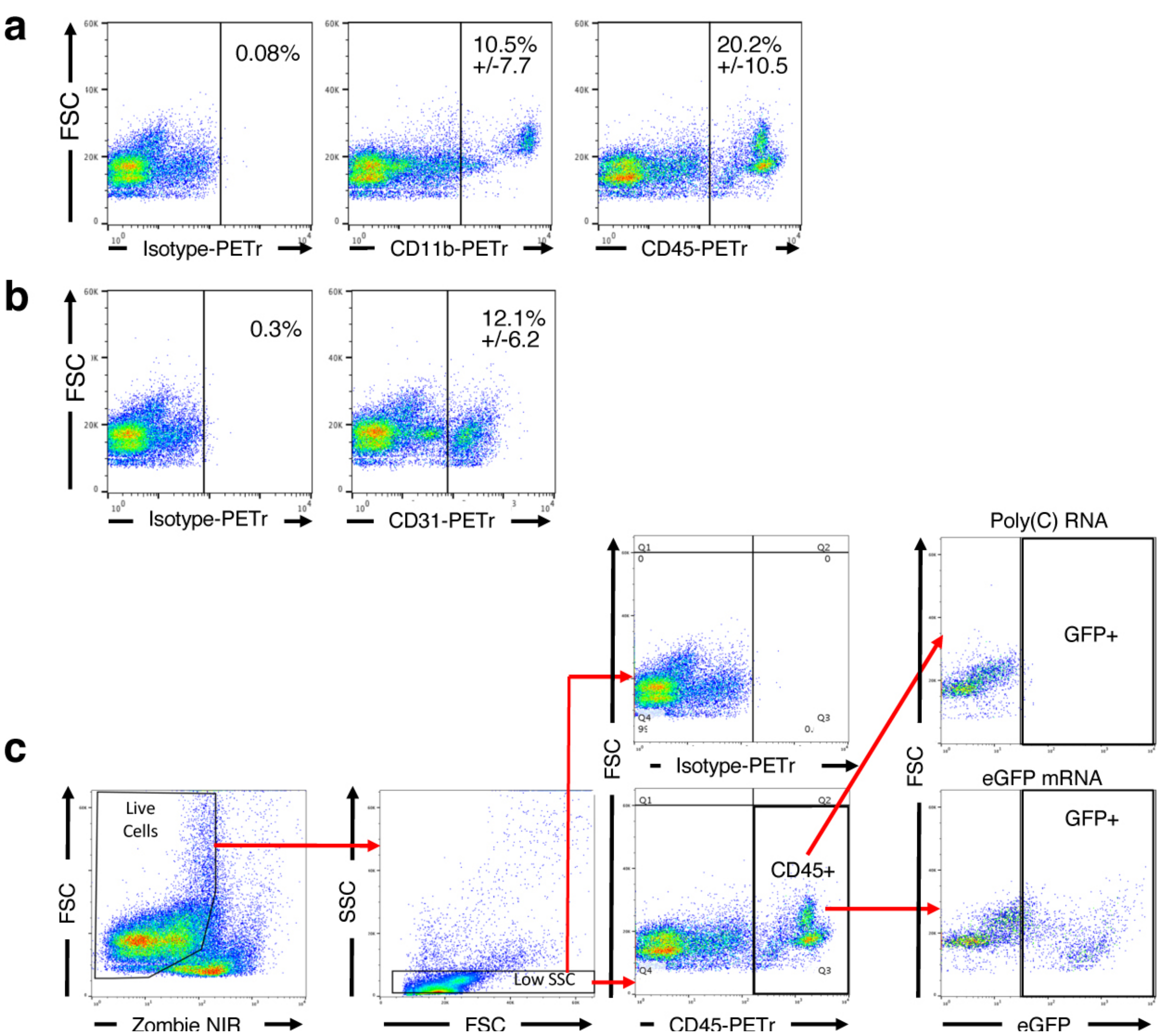

Supplementary Fig. 5 Quantification of endothelial and blood cells from liver NPC fractions from mice injected IV with mRNA-LNP. **a, b** Representative flow cytometry analyses of NPC fractions from mice 5 hours after a single injection of Poly(C) RNA-LNP (n=3 mice) or eGFP mRNA-LNP (n=3 mice) are shown. Plots represent the percent of cells positive for CD11b, CD45 (**a**), and CD31 (**b**) gated from live cell population (Zombie NIR-negative NPC fraction) compared to isotypes for each antibody (rat IgG2b kappa for CD11b and CD45; rat IgG2a kappa for CD31). Data are presented as mean values +/- SD for n=3 mice per group. **c** Representative gating strategy from CD45 staining to isolate live GFP+ CD45+ cells as compared to isotype control and Poly (C) RNA-injected cells.

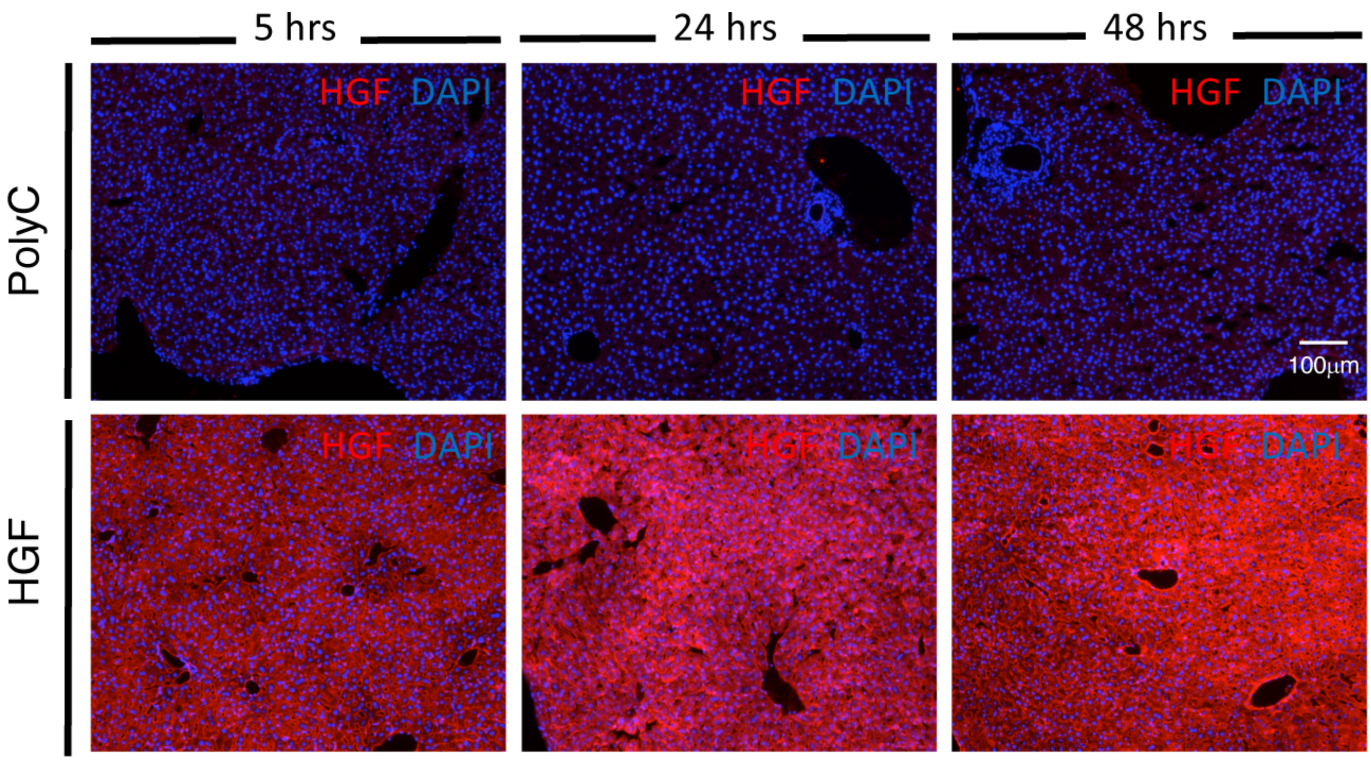

Supplementary Fig. 6 Efficient expression of HGF protein following a single injection of HGF mRNA-LNP. Immunostaining for HGF on liver sections 5, 24 and 48 hours after a single injection of Poly(C) RNA-LNP or HGF mRNA-LNP. 100X magnification pictures are shown. Representative images from 2 mice injected with either Poly(C) mRNA-LNP or HGF mRNA-LNP per time point. The scale bar represents 100μm for all images.

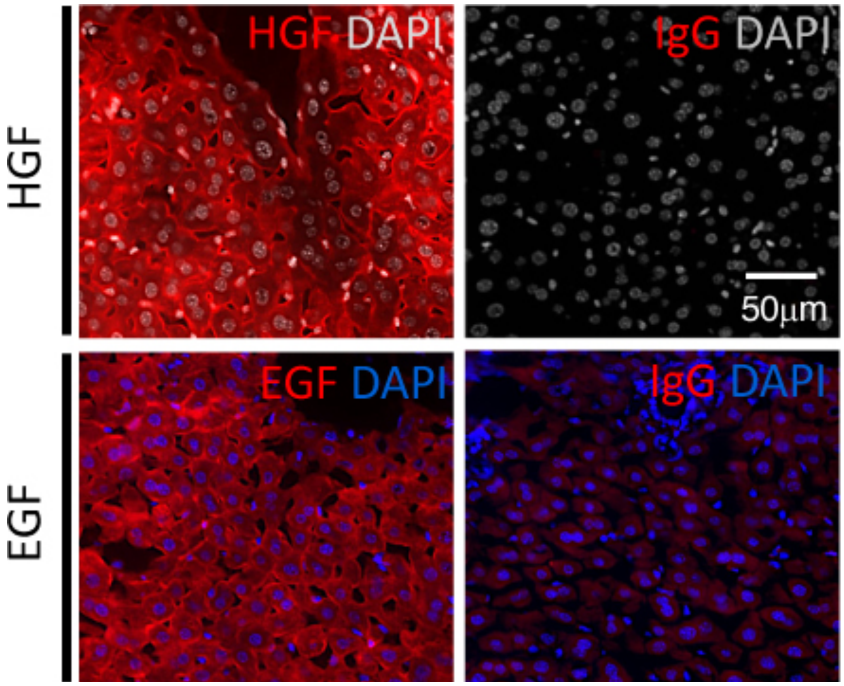

Supplementary Fig. 7 Efficient expression of HGF and EGF proteins via injection of the corresponding mRNA-LNP into uninjured mice. Mice were injected with a single dose of HGF or EGF mRNA-LNP and liver sections analyzed 24 hours after injection. Control IgG are shown for each specific staining. 200X magnification pictures are shown. Representative images from 3 mice injected with either HGF mRNA-LNP or EGF mRNA-LNP. The scale bar represents 50μm for all 200X magnification images.

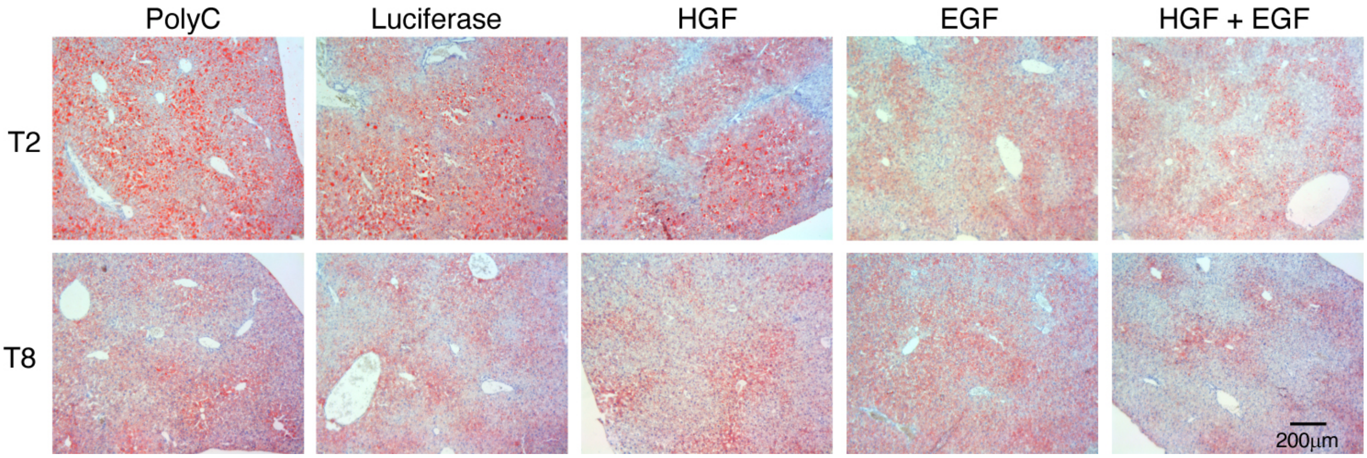

Supplementary Fig. 8 Injection of both HGF/EGF mRNA-LNP decrease the lipid content in hepatocytes following CDE diet. Mice were fed CDE diet for 3 weeks as explained in Fig. 4A and then injected with a single dose of HGF or EGF mRNA-LNP or combined together, while Poly(C) RNA-LNP and Luc mRNA-LNP were used as negative controls. Liver sections were stained with Oil Red O to detect the lipid content in hepatocytes at T2 and T8, 2 and 8 days after the diet was discontinued. Representative pictures obtained for 4 mice per group per time point are shown (100X magnification). The scale bar represents 200μm for all 100X magnification images.

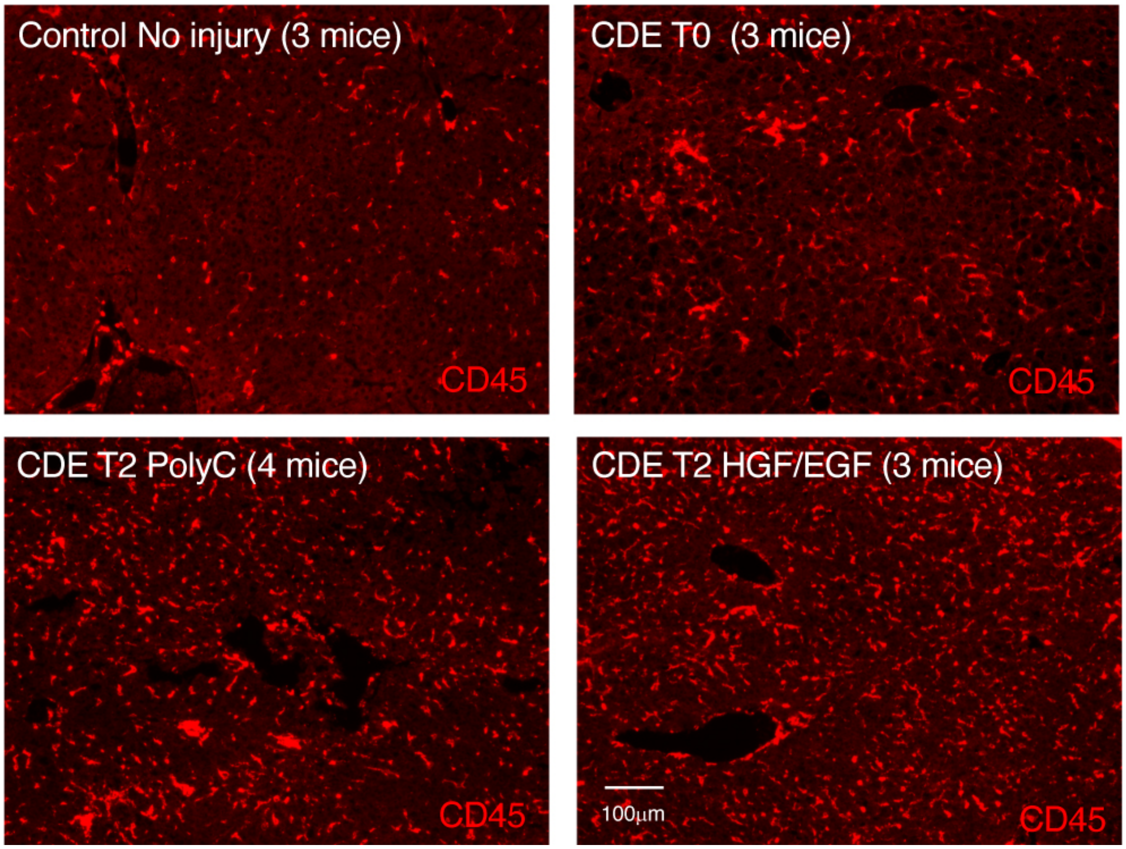

Supplementary Fig. 9 RNA-LNP treatment induces equally a mild inflammation in control Poly(C) RNA-LNP- and HGF/EGF mRNA-LNP- treated mice. Mice were fed normal diet (control No injury) or CDE diet for 3 weeks as explained in Fig. 4A and then injected with a single dose of HGF/EGF mRNA-LNP or Poly(C) RNA-LNP. Liver sections were immunostained for CD45 at T0 and T2, 0 and 2 days after the diet was discontinued. Representative pictures obtained for 3-4 mice per group per time point are shown (100X magnification). The scale bar represents 100μm for all images.

B. Supplementary Tables

Supplementary Table 1: List of antibodies

Antibody	Cat. No	Species	Application	Dilution
GFP	A10262 (Invitrogen)	Chicken	IHC-Fr	1:300
HNF4α	sc-6556 (Santa Cruz)	Goat	IHC-Fr	1:50
CK19	602-670 (Abbomax)	Rabbit	IHC-Fr	1:100
CD31	557355 (BD Pharmingen)	Rat	IHC-Fr	1:100
CD45	NB100-77417 (Novus)	Rat	IHC-Fr	1:100
α-SMA	19245S (Cell Signaling)	Rabbit	IHC-Fr	1:300
F4/80	sc25830 (Santa Cruz)	Rabbit	IHC-Fr	1:100
HGF	AF-294-NA (R&D)	Goat	IHC-Fr	1:20
EGF	MAB236-100	Mouse	IHC-Fr	1:30
CD31-PE/Dazzle 594	102429 (BioLegend)	Rat	FC	1:40
CD11b-PE efluor 610	61-0112-80 (Invitrogen)	Rat	FC	1:80
CD45-PE efluor 610	61-0451-80 (Invitrogen)	Rat	FC	1:40

IHC-Fr: Immunohistochemistry frozen fixed tissues; FC: Flow Cytometry

Supplementary Table 2: Sequences of nucleoside-modified mRNA

**Luciferase**	ATGGAGGACGCCAAGAACATCAAGAAGGGCCCCGCCCCCTTCTACCCCCTGGAGGACGGCACCGCCGGCGAGCAGCTGCACAAGGCC<br>ATGAAGCGgTACGCCCTGGTGCCCGGCACCATCGCCTTCACCGACGCCCACATCGAGGTGGACATCACCTACGCCGAGTACTTCGAGAT<br>GTCCGTGCGCCTGGCCGAGGCCATGAAGCGgTACGGCCTGAACACCAACCACCGCATCGTGGTGTGCTCCGAGAACTCCCTGCAGTTCT<br>TCATGCCCGTGCTGGGCGCCCTGTTCATCGGCGTGGCCGTGGCCCCCGCCAACGACATCTACAACGAGCGCGAGCTGCTGAACTCCATG<br>GGCATCTCCCAGCCCACCGTGGTGTTCGTGTCCAAGAAGGGCCTGCAGAAGATCCTGAACGTGCAGAAGAAGCTGCCCATCATCCAGA<br>AGATCATCATCATGGACTCCAAGACCGACTACCAGGGCTTCCAGTCCATGTACACCTTCGTGACCTCCCACCTGCCCCCCGGCTTCAACG<br>AGTACGACTTCGTGCCCGAGTCCTTCGACCGCGACAAGACCATCGCCCTGATCATGAACTCCTCCGGCTCCACCGGCCTGCCCAAGGGC<br>GTGGCCCTGCCCCACCGCACCGCCTGCGTGCGCTTCTCCCACGCCCGCGACCCCATCTTCGGCAACCAGATCATCCCCGACACCGCCATC<br>CTGTCCGTGGTGCCCTTCCACCACGGCTTCGGCATGTTCACCACCCTGGGCTACCTGATCTGCGGCTTCCGCGTGGTGCTGATGTACCGC<br>TTCGAGGAGGAGCTGTTCCTGCGCTCCCTGCAGGACTACAAGATCCAGTCCGCCCTGCTGGTGCCCACCCTGTTCTCCTTCTTCGCCAAG<br>TCCACCCTGATCGACAAGTACGACCTGTCCAACCTGCACGAGATCGCCTCCGGCGGCGCCCCCCTGTCCAAGGAGGTGGGCGAGGCCG<br>TGGCCAAGCGgTTCCACCTGCCCGGCATCCGCCAGGGCTACGGCCTGACCGAGACCACCTCCGCCATCCTGATCACCCCCGAGGGCGAC<br>GACAAGCCCGGCGCCGTGGGCAAGGTGGTGCCCTTCTTCGAGGCCAAGGTGGTGGACCTGGACACCGGCAAGACCCTGGGCGTGAAC<br>CAGCGCGGCGAGCTGTGCGTGCGCGGCCCCATGATCATGTCCGGCTACGTGAACAACCCCGAGGCCACCAACGCCCTGATCGACAAGG<br>ACGGCTGGCTGCACTCCGGCGACATCGCCTACTGGGACGAGGACGAGCACTTCTTCATCGTGGACCGCCTGAAGTCCCTGATCAAGTAC<br>AAGGGCTACCAGGTGGCCCCCGCCGAGCTGGAGTCCATCCTGCTGCAGCACCCCAACATCTTCGACGCCGGCGTGGCCGGCCTGCCCG<br>ACGACGACGCCGGCGAGCTGCCCGCCGCCGTGGTGGTGCTGGAGCACGGCAAGACCATGACCGAGAAGGAGATCGTGGACTACGTGG<br>CCTCCCAGGTGACCACCGCCAAGAAGCTGCGCGGCGGCGTGGTGTTCGTGGACGAGGTGCCCAAGGGCCTGACCGGCAAGCTGGACG<br>CCCGCAAGATCCGCGAGATCCTGATCAAGGCCAAGAAGGGCGGCAAGATCGCCGTG
**eGFP**	ATGGTGAGCAAGGGCGAGGAGCTGTTCACCGGGGTGGTGCCCATCCTGGTCGAGCTGGACGGCGACGTAAACGGCCACAAGTTCAGC<br>GTGTCCGGCGAGGGCGAGGGCGATGCCACCTACGGCAAGCTGACCCTGAAGTTCATCTGCACCACCGGCAAGCTGCCCGTGCCCTGGC<br>CCACCCTCGTGACCACCCTGACCTACGGCGTGCAGTGCTTCAGCCGCTACCCCGACCACATGAAGCAGCACGACTTCTTCAAGTCCGCCA<br>TGCCCGAAGGCTACGTCCAGGAGCGCACCATCTTCTTCAAGGACGACGGCAACTACAAGACCCGCGCCGAGGTGAAGTTCGAGGGCGA<br>CACCCTGGTGAACCGCATCGAGCTGAAGGGCATCGACTTCAAGGAGGACGGCAACATCCTGGGGCACAAGCTGGAGTACAACTACAAC<br>AGCCACAACGTCTATATCATGGCCGACAAGCAGAAGAACGGCATCAAGGTGAACTTCAAGATCCGCCACAACATCGAGGACGGCAGCG<br>TGCAGCTCGCCGACCACTACCAGCAGAACACCCCCATCGGCGACGGCCCCGTGCTGCTGCCCGACAACCACTACCTGAGCACCCAGTCC<br>GCCCTGAGCAAAGACCCCAACGAGAAGCGCGATCACATGGTCCTGCTGGAGTTCGTGACCGCCGCCGGGATCACTCTCGGCATGGACG<br>AGCTGTACAAG
**HGF**	ATGTGGGTGACCAAGCTGCTGCCCGCCCTGCTGCTGCAGCACGTGCTGCTGCACCTGCTGCTGCTGCCCATCGCCATCCCCTACGCCGA<br>GGGCCAGCGCAAGCGCCGCAACACCATCCACGAGTTCAAGAAGTCCGCCAAGACCACCCTGATCAAGATCGACCCCGCCCTGAAGATC<br>AAGACCAAGAAGGTGAACACCGCCGACCAGTGCGCCAACCGCTGCACCCGCAACAAGGGCCTGCCCTTCACCTGCAAGGCCTTCGTGTT<br>CGACAAGGCCCGCAAGCAGTGCCTGTGGTTCCCCTTCAACTCCATGTCCTCCGGCGTGAAGAAGGAGTTCGGCCACGAGTTCGACCTGT

	ACGAGAACAAGGACTACATCCGCAACTGCATCATCGGCAAGGGCCGCTCCTACAAGGGCACCGTGTCCATCACCAAGTCCGGCATCAA<br>GTGCCAGCCCTGGTCCTCCATGATCCCCCACGAGCACTCCTTCCTGCCCTCCTCCTACCGCGGCAAGGACCTGCAGGAGAACTACTGCCG<br>CAACCCCCGCGGCGAGGAGGGCGGCCCCTGGTGCTTCACCTCCAACCCCGAGGTGCGCTACGAGGTGTGCGACATCCCCCAGTGCTCC<br>GAGGTGGAGTGCATGACCTGCAACGGCGAGTCCTACCGCGGCCTGATGGACCACACCGAGTCCGGCAAGATCTGCCAGCGgTGGGACC<br>ACCAGACCCCCCACCGCCACAAGTTCCTGCCCGAGCGgTACCCCGACAAGGGCTTCGACGACAACTACTGCCGCAACCCCGACGGCCAG<br>CCCCGCCCCTGGTGCTACACCCTGGACCCCCACACCCGCTGGGAGTACTGCGCCATCAAGACCTGCGCCGACAACACCATGAACGACAC<br>CGACGTGCCCCTGGAGACCACCGAGTGCATCCAGGGCCAGGGCGAGGGCTACCGCGGCACCGTGAACACCATCTGGAACGGCATCCCC<br>TGCCAGCGgTGGGACTCCCAGTACCCCCACGAGCACGACATGACCCCCGAGAACTTCAAGTGCAAGGACCTGCGCGAGAACTACTGCC<br>GCAACCCCGACGGCTCCGAGTCCCCCTGGTGCTTCACCACCGACCCCAACATCCGCGTGGGCTACTGCTCCCAGATCCCCAACTGCGACA<br>TGTCCCACGGCCAGGACTGCTACCGCGGCAACGGCAAGAACTACATGGGCAACCTGTCCCAGACCCGCTCCGGCCTGACCTGCTCCATG<br>TGGGACAAGAACATGGAGGACCTGCACCGCCACATCTTCTGGGAGCCCGACGCCTCCAAGCTGAACGAGAACTACTGCCGCAACCCCG<br>ACGACGACGCCCACGGCCCCTGGTGCTACACCGGCAACCCCCTGATCCCCTGGGACTACTGCCCCATCTCCCGCTGCGAGGGCGACACC<br>ACCCCCACCATCGTGAACCTGGACCACCCCGTGATCTCCTGCGCCAAGACCAAGCAGCTGCGCGTGGTGAACGGCATCCCCACCCGCAC<br>CAACATCGGCTGGATGGTGTCCCTGCGCTACCGCAACAAGCACATCTGCGGCGGCTCCCTGATCAAGGAGTCCTGGGTGCTGACCGCCC<br>GCCAGTGCTTCCCCTCCCGCGACCTGAAGGACTACGAGGCCTGGCTGGGCATCCACGACGTGCACGGCCGCGGCGACGAGAAGTGCAA<br>GCAGGTGCTGAACGTGTCCCAGCTGGTGTACGGCCCCGAGGGCTCCGACCTGGTGCTGATGAAGCTGGCCCGCCCCGCCGTGCTGGAC<br>GACTTCGTGTCCACCATCGACCTGCCCAACTACGGCTGCACCATCCCCGAGAAGACCTCCTGCTCCGTGTACGGCTGGGGCTACACCGG<br>CCTGATCAACTACGACGGCCTGCTGCGCGTGGCCCACCTGTACATCATGGGCAACGAGAAGTGCTCCCAGCACCACCGCGGCAAGGTG<br>ACCCTGAACGAGTCCGAGATCTGCGCCGGCGCCGAGAAGATCGGCTCCGGCCCCTGCGAGGGCGACTACGGCGGCCCCCTGGTGTGC<br>GAGCAGCACAAGATGCGCATGGTGCTGGGCGTGATCGTGCCCGGCCGCGGCTGCGCCATCCCCAACCGCCCCGGCATCTTCGTGCGCG<br>TGGCCTACTACGCCAAGTGGATCCACAAGATCATCCTGACCTACAAGGTGCCCCAGTCC
**EGF**	ATGCTGCTGACCCTGATCATCCTGCTGCCCGTGGTGTCCAAGTTCTCCTTCGTGTCCCTGTCCGCCCCCCAGCACTGGTCCTGCCCCGAGG<br>GCACCCTGGCCGGCAACGGCAACTCCACCTGCGTGGGCCCCGCCCCCTTCCTGATCTTCTCCCACGGCAACTCCATCTTCCGCATCGACA<br>CCGAGGGCACCAACTACGAGCAGCTGGTGGTGGACGCCGGCGTGTCCGTGATCATGGACTTCCACTACAACGAGAAGCGCATCTACTG<br>GGTGGACCTGGAGCGCCAGCTGCTGCAGCGCGTGTTCCTGAACGGCTCCCGCCAGGAGCGCGTGTGCAACATCGAGAAGAACGTGTCC<br>GGCATGGCCATCAACTGGATCAACGAGGAGGTGATCTGGTCCAACCAGCAGGAGGGCATCATCACCGTGACCGACATGAAGGGCAAC<br>AACTCCCACATCCTGCTGTCCGCCCTGAAGTACCCCGCCAACGTGGCCGTGGACCCCGTGGAGCGgTTCATCTTCTGGTCCTCCGAGGTG<br>GCCGGCTCCCTGTACCGCGCCGACCTGGACGGCGTGGGCGTGAAGGCCCTGCTGGAGACCTCCGAGAAGATCACCGCCGTGTCCCTGG<br>ACGTGCTGGACAAGCGCCTGTTCTGGATCCAGTACAACCGCGAGGGCTCCAACTCCCTGATCTGCTCCTGCGACTACGACGGCGGCTCC<br>GTGCACATCTCCAAGCACCCCACCCAGCACAACCTGTTCGCCATGTCCCTGTTCGGCGACCGCATCTTCTACTCCACCTGGAAGATGAAG<br>ACCATCTGGATCGCCAACAAGCACACCGGCAAGGACATGGTGCGCATCAACCTGCACTCCTCCTTCGTGCCCCTGGGCGAGCTGAAGGT<br>GGTGCACCCCCTGGCCCAGCCCAAGGCCGAGGACGACACCTGGGAGCCCGAGCAGAAGCTGTGCAAGCTGCGCAAGGGCAACTGCTC<br>CTCCACCGTGTGCGGCCAGGACCTGCAGTCCCACCTGTGCATGTGCGCCGAGGGCTACGCCCTGTCCCGCGACCGCAAGTACTGCGAG<br>GACGTGAACGAGTGCGCCTTCTGGAACCACGGCTGCACCCTGGGCTGCAAGAACACCCCCGGCTCCTACTACTGCACCTGCCCCGTGGG<br>CTTCGTGCTGCTGCCCGACGGCAAGCGgTGCCACCAGCTGGTGTCCTGCCCCCGCAACGTGTCCGAGTGCTCCCACGACTGCGTGCTGA<br>CCTCCGAGGGCCCCCTGTGCTTCTGCCCCGAGGGCTCCGTGCTGGAGCGCGACGGCAAGACCTGCTCCGGCTGCTCCTCCCCCGACAAC<br>GGCGGCTGCTCCCAGCTGTGCGTGCCCCTGTCCCCCGTGTCCTGGGAGTGCGACTGCTTCCCCGGCTACGACCTGCAGCTGGACGAGAA<br>GTCCTGCGCCGCCTCCGGCCCCCAGCCCTTCCTGCTGTTCGCCAACTCCCAGGACATCCGCCACATGCACTTCGACGGCACCGACTACGG<br>CACCCTGCTGTCCCAGCAGATGGGCATGGTGTACGCCCTGGACCACGACCCCGTGGAGAACAAGATCTACTTCGCCCACACCGCCCTGA<br>AGTGGATCGAGCGCGCCAACATGGACGGCTCCCAGCGCGAGCGCCTGATCGAGGAGGGCGTGGACGTGCCCGAGGGCCTGGCCGTG<br>GACTGGATCGGCCGCCGCTTCTACTGGACCGACCGCGGCAAGTCCCTGATCGGCCGCTCCGACCTGAACGGCAAGCGgTCCAAGATCAT<br>CACCAAGGAGAACATCTCCCAGCCCCGCGGCATCGCCGTGCACCCCATGGCCAAGCGCCTGTTCTGGACCGACACCGGCATCAACCCCC<br>GCATCGAGTCCTCCTCCCTGCAGGGCCTGGGCCGCCTGGTGATCGCCTCCTCCGACCTGATCTGGCCCTCCGGCATCACCATCGACTTCC<br>TGACCGACAAGCTGTACTGGTGCGACGCCAAGCAGTCCGTGATCGAGATGGCCAACCTGGACGGCTCCAAGCGCCGCCGCCTGACCCA<br>GAACGACGTGGGCCACCCCTTCGCCGTGGCCGTGTTCGAGGACTACGTGTGGTTCTCCGACTGGGCCATGCCCTCCGTGATCCGCGTGA<br>ACAAGCGCACCGGCAAGGACCGCGTGCGCCTGCAGGGCTCCATGCTGAAGCCCTCCTCCCTGGTGGTGGTGCACCCCCTGGCCAAGCC<br>CGGCGCCGACCCCTGCCTGTACCAGAACGGCGGCTGCGAGCACATCTGCAAGAAGCGCCTGGGCACCGCCTGGTGCTCCTGCCGCGAG<br>GGCTTCATGAAGGCCTCCGACGGCAAGACCTGCCTGGCCCTGGACGGCCACCAGCTGCTGGCCGGCGGCGAGGTGGACCTGAAGAAC<br>CAGGTGACCCCCCTGGACATCCTGTCCAAGACCCGCGTGTCCGAGGACAACATCACCGAGTCCCAGCACATGCTGGTGGCCGAGATCAT<br>GGTGTCCGACCAGGACGACTGCGCCCCCGTGGGCTGCTCCATGTACGCCCGCTGCATCTCCGAGGGCGAGGACGCCACCTGCCAGTGC<br>CTGAAGGGCTTCGCCGGCGACGGCAAGCTGTGCTCCGACATCGACGAGTGCGAGATGGGCGTGCCCGTGTGCCCCCCCGCCTCCTCCA<br>AGTGCATCAACACCGAGGGCGGCTACGTGTGCCGCTGCTCCGAGGGCTACCAGGGCGACGGCATCCACTGCCTGGACATCGACGAGTG<br>CCAGCTGGGCGTGCACTCCTGCGGCGAGAACGCCTCCTGCACCAACACCGAGGGCGGCTACACCTGCATGTGCGCCGGCCGCCTGTCC<br>GAGCCCGGCCTGATCTGCCCCGACTCCACCCCCCCCCCCCACCTGCGCGAGGACGACCACCACTACTCCGTGCGCAACTCCGACTCCGAG<br>TGCCCCCTGTCCCACGACGGCTACTGCCTGCACGACGGCGTGTGCATGTACATCGAGGCCCTGGACAAGTACGCCTGCAACTGCGTGGT<br>GGGCTACATCGGCGAGCGgTGCCAGTACCGCGACCTGAAGTGGTGGGAGCTGCGCCACGCCGGCCACGGCCAGCAGCAGAAGGTGAT<br>CGTGGTGGCCGTGTGCGTGGTGGTGCTGGTGATGCTGCTGCTGCTGTCCCTGTGGGGCGCCCACTACTACCGCACCCAGAAGCTGCTGT<br>CCAAGAACCCCAAGAACCCCTACGAGGAGTCCTCCCGCGACGTGCGCTCCCGCCGCCCCGCCGACACCGAGGACGGCATGTCCTCCTGC<br>CCCCAGCCCTGGTTCGTGGTGATCAAGGAGCACCAGGACCTGAAGAACGGCGGCCAGCCCGTGGCCGGCGAGGACGGCCAGGCCGCC<br>GACGGCTCCATGCAGCCCACCTCCTGGCGCCAGGAGCCCCAGCTGTGCGGCATGGGCACCGAGCAGGGCTGCTGGATCCCCGTGTCCT<br>CCGACAAGGGCTCCTGCCCCCAGGTGATGGAGCGgTCCTTCCACATGCCCTCCTACGGCACCCAGACCCTGGAGGGCGGCGTGGAGAA<br>GCCCCACTCCCTGCTGTCCGCCAACCCCCTGTGGCAGCAGCGCGCCCTGGACCCCCCCCACCAGATGGAGCTGACCCAG
