# Supplementary material for: Murine liver repair via transient activation of regenerative pathways in hepatocytes using lipid nanoparticle-complexed nucleoside-modified mRNA
Source: Nat Commun. 2021 Jan 27;12:613. doi: 10.1038/s41467-021-20903-3 (PMC7840919; doi:10.1038/s41467-021-20903-3)
Supplement: Supplementary file 3 — Reporting Summary [file 41467_2021_20903_MOESM3_ESM.pdf]

nature research

Corresponding author(s): Valerie Gouon-Evans
Last updated by author(s): 2020.12.15

Reporting Summary

Nature Research wishes to improve the reproducibility of the work that we publish. This form provides structure for consistency and transparency in reporting. For further information on Nature Research policies, see our Editorial Policies and the Editorial Policy Checklist.

Statistics

For all statistical analyses, confirm that the following items are present in the figure legend, table legend, main text, or Methods section.

n/a	Confirmed	
☐	☒	The exact sample size (*n*) for each experimental group/condition, given as a discrete number and unit of measurement
☐	☒	A statement on whether measurements were taken from distinct samples or whether the same sample was measured repeatedly
☐	☒	The statistical test(s) used AND whether they are one- or two-sided<br>*Only common tests should be described solely by name; describe more complex techniques in the Methods section.*
☐	☒	A description of all covariates tested
☒	☐	A description of any assumptions or corrections, such as tests of normality and adjustment for multiple comparisons
☐	☒	A full description of the statistical parameters including central tendency (e.g. means) or other basic estimates (e.g. regression coefficient) AND variation (e.g. standard deviation) or associated estimates of uncertainty (e.g. confidence intervals)
☒	☐	For null hypothesis testing, the test statistic (e.g. *F*, *t*, *r*) with confidence intervals, effect sizes, degrees of freedom and *P* value noted<br>*Give P values as exact values whenever suitable.*
☒	☐	For Bayesian analysis, information on the choice of priors and Markov chain Monte Carlo settings
☒	☐	For hierarchical and complex designs, identification of the appropriate level for tests and full reporting of outcomes
☒	☐	Estimates of effect sizes (e.g. Cohen's *d*, Pearson's *r*), indicating how they were calculated

Our web collection on statistics for biologists contains articles on many of the points above.

Software and code

Policy information about availability of computer code

Data collection	The statistical analyses were carried out using Student's t test for unpaired comparisons. A p value less than 0.05 was considered significant. The results are presented as the means ± standard error (SEM) or standard deviation (SD) (for flow cytometry data only) as indicated in each figure legend. n.s.: non-significant p>0.05, * p<0.05, ** p<0.001, *** p<0.0001. Excel was used to calculate statistics.
Data analysis	We used with FlowJo v10 software to analyze flow cytometry data.

For manuscripts utilizing custom algorithms or software that are central to the research but not yet described in published literature, software must be made available to editors and reviewers. We strongly encourage code deposition in a community repository (e.g. GitHub). See the Nature Research guidelines for submitting code & software for further information.

Data

Policy information about availability of data

All manuscripts must include a data availability statement. This statement should provide the following information, where applicable:

- Accession codes, unique identifiers, or web links for publicly available datasets
- A list of figures that have associated raw data
- A description of any restrictions on data availability

All data generated or analyzed during this study are included in this published article and its supplementary information. These include raw data for graphs related to Figures 1B,2B, 3E, 4C, 4D, 5C, 5D, 6C and 6E are also included in supplementary information and Source Data file.

Field-specific reporting

Please select the one below that is the best fit for your research. If you are not sure, read the appropriate sections before making your selection.

- [x] Life sciences
- [ ] Behavioural & social sciences
- [ ] Ecological, evolutionary & environmental sciences

For a reference copy of the document with all sections, see nature.com/documents/nr-reporting-summary-flat.pdf

Life sciences study design

All studies must disclose on these points even when the disclosure is negative.

Sample size	We used mostly 3-4 mice per group tested in each experiment based on previous publications using the same injury models and assays to characterized the liver injury. The sample size was shown to be sufficient to identify statistical differences between groups for the specific assays used. All group sizes are indicated in each figure legends.
Data exclusions	No data were excluded
Replication	All in vivo experiments were performed with 3-4 mice per group and per time point or otherwise indicated in the figure legends. Histology of all lobes of each mouse livers were analyzed and 2-4 pictures taken to represent each liver.
Randomization	Mice used in this study were inbred, all the same age, the same sex, and therefore about the same weight. Mice were provided by the Jackson laboratory.
Blinding	All tissue staining were analyzed by 4 blinded authors (the first 3 authors as well as the last author). Liver tissue staining or immunostaining-based data were quantified and supported by analyses of serum data from the same mice.

Reporting for specific materials, systems and methods

We require information from authors about some types of materials, experimental systems and methods used in many studies. Here, indicate whether each material, system or method listed is relevant to your study. If you are not sure if a list item applies to your research, read the appropriate section before selecting a response.

Materials & experimental systems

n/a	Involved in the study
[ ]	[x] Antibodies
[ ]	[ ] Eukaryotic cell lines
[ ]	[ ] Palaeontology and archaeology
[ ]	[x] Animals and other organisms
[ ]	[ ] Human research participants
[ ]	[ ] Clinical data
[ ]	[ ] Dual use research of concern

Methods

n/a	Involved in the study
[ ]	[ ] ChIP-seq
[ ]	[x] Flow cytometry
[ ]	[ ] MRI-based neuroimaging

Antibodies

Antibodies used	All antibodies used in this study have been listed in the table in the supplementary data file.
Validation	We validated specificity of each antibody for immuno-staining by using the corresponding isotype negative controls.

Eukaryotic cell lines

Policy information about cell lines

Cell line source(s)	*State the source of each cell line used.*
Authentication	*Describe the authentication procedures for each cell line used OR declare that none of the cell lines used were authenticated.*
Mycoplasma contamination	*Confirm that all cell lines tested negative for mycoplasma contamination OR describe the results of the testing for mycoplasma contamination OR declare that the cell lines were not tested for mycoplasma contamination.*
Commonly misidentified lines (See ICLAC register)	*Name any commonly misidentified cell lines used in the study and provide a rationale for their use.*

Palaeontology and Archaeology

Specimen provenance	*Provide provenance information for specimens and describe permits that were obtained for the work (including the name of the issuing authority, the date of issue, and any identifying information).*
Specimen deposition	*Indicate where the specimens have been deposited to permit free access by other researchers.*
Dating methods	*If new dates are provided, describe how they were obtained (e.g. collection, storage, sample pretreatment and measurement), where they were obtained (i.e. lab name), the calibration program and the protocol for quality assurance OR state that no new dates are provided.*

☐ Tick this box to confirm that the raw and calibrated dates are available in the paper or in Supplementary Information.

Ethics oversight	*Identify the organization(s) that approved or provided guidance on the study protocol, OR state that no ethical approval or guidance was required and explain why not.*

Note that full information on the approval of the study protocol must also be provided in the manuscript.

Animals and other organisms

Policy information about studies involving animals; ARRIVE guidelines recommended for reporting animal research

Laboratory animals	All mice were inbred female Black6C57.Blc from the Jackson Laboratory. Their age varied between 10-12 weeks of age.
Wild animals	N/A
Field-collected samples	N/A
Ethics oversight	All studies with mice have been approved by the BU BMC AICUC

Note that full information on the approval of the study protocol must also be provided in the manuscript.

Human research participants

Policy information about studies involving human research participants

Population characteristics	N/A
Recruitment	N/A
Ethics oversight	N/A

Note that full information on the approval of the study protocol must also be provided in the manuscript.

Clinical data

Policy information about clinical studies

All manuscripts should comply with the ICMJEguidelines for publication of clinical research and a completedCONSORT checklist must be included with all submissions.

Clinical trial registration	N/A
Study protocol	N/A
Data collection	N/A
Outcomes	N/A

Dual use research of concern

Policy information about dual use research of concern

Hazards

Could the accidental, deliberate or reckless misuse of agents or technologies generated in the work, or the application of information presented in the manuscript, pose a threat to:

No	Yes	
☒	☐	Public health
☒	☐	National security
☒	☐	Crops and/or livestock
☒	☐	Ecosystems
☒	☐	Any other significant area

Experiments of concern

Does the work involve any of these experiments of concern:

No	Yes	
☒	☐	Demonstrate how to render a vaccine ineffective
☒	☐	Confer resistance to therapeutically useful antibiotics or antiviral agents
☒	☐	Enhance the virulence of a pathogen or render a nonpathogen virulent
☒	☐	Increase transmissibility of a pathogen
☒	☐	Alter the host range of a pathogen
☒	☐	Enable evasion of diagnostic/detection modalities
☒	☐	Enable the weaponization of a biological agent or toxin
☒	☐	Any other potentially harmful combination of experiments and agents

ChIP-seq

Data deposition

☐ Confirm that both raw and final processed data have been deposited in a public database such as GEO.

☐ Confirm that you have deposited or provided access to graph files (e.g. BED files) for the called peaks.

Data access links<br>*May remain private before publication.*	N/A
Files in database submission	N/A
Genome browser session<br>(e.g. UCSC)	N/A

Methodology

Replicates	N/A
Sequencing depth	N/A
Antibodies	N/A
Peak calling parameters	N/A
Data quality	N/A
Software	N/A

Flow Cytometry

Plots

Confirm that:

☒ The axis labels state the marker and fluorochrome used (e.g. CD4-FITC).

☒ The axis scales are clearly visible. Include numbers along axes only for bottom left plot of group (a 'group' is an analysis of identical markers).

☒ All plots are contour plots with outliers or pseudocolor plots.

☒ A numerical value for number of cells or percentage (with statistics) is provided.

Methodology

Sample preparation	Liver was perfused according to the previously published method with minor modifications54. All solutions were pre-warmed to 40°C and delivered at a rate of 4 ml/min. Mice were anaesthetized with ketamine/xylazine and then perfused by cannulation with a 24-gauge catheter through the inferior vena cava with 30mL 1X Liver Perfusion Medium (Gibco by Life Technologies), while the portal vein was cut to allow the perfusate to flow out. 15 mL of Earle's Balanced Salt Solution (EBSS) containing 10 mM Hepes (Ca^{++} and Mg^{++}, pH 7.4) was then perfused, and finally followed with 30 mL of Liver Digest Medium (Gibco by Life Technologies) to allow complete dissociation of liver cells in situ. Livers were then extracted and mechanically dissociated in 10mL of Liver Digest Medium. Cells were filtered through 100 µm cell strainer and filtrate was centrifuged at 50 x g for 2 min at 4°C to obtain hepatocytes in the pellet while the supernatant was collected as the fraction of non-parenchymal cells (NPCs). The hepatocyte pellet was resuspended in wash media (Hepatocyte Wash Medium by Gibco + 0.1mg/mL DNAse 1 + 10% FBS). The cell fraction caught in the 100 µm strainer was further digested in NPC Digest Medium (2.5 mg/mL Collagenase IV + 0.1mg/mL DNAse 1), filtered through a 40 µm strainer, then centrifuged at 300 x g for 5 minutes at 4°C. The supernatant was discarded, pellet was resuspended in wash media, and solution was added to the other collection of NPCs. Following 10-minute incubations in wash media, both fractions were washed then resuspended in PBS. Fractions were incubated in Zombie NIR Fixable Viability dye (1:2000, Biolegend # 423105) for 30 minutes at room temperature, washed, and resuspended in FACS buffer (2% FBS + 2mM EDTA at pH 8). Fractions were then separated into 100 µl wells and treated with 1 µg/100 µl Fc Block (BD Pharmingen #553141) for 10 minutes at room temperature. Wells were then incubated with specific antibodies for 20 minutes at room temperature. Following antibody incubation, cells were washed and resuspended in FACS buffer. Cells were run on a Stratedigm S1000EXi flow cytometer and data analyzed with FlowJo v10 software. Care was taken to include proper unstained and isotype controls to derive the compensation matrix.
Instrument	Stratedigm S1000EXi flow cytometer
Software	FlowJo v10 software
Cell population abundance	we collected at least 20,000 cells
Gating strategy	Cells were first gated using the SSC and FSC parameters to help eliminate debris. Dead cells were eliminated using the Zombie NIR viability die. The remaining cells were analyzed for receptor expression.

☒ Tick this box to confirm that a figure exemplifying the gating strategy is provided in the Supplementary Information.

Magnetic resonance imaging

Experimental design

Design type	N/A
Design specifications	N/A
Behavioral performance measures	N/A

Acquisition

Imaging type(s)	N/A
Field strength	N/A
Sequence & imaging parameters	N/A
Area of acquisition	*State whether a whole brain scan was used OR define the area of acquisition, describing how the region was determined.*

Diffusion MRI ☐ Used ☒ Not used

Preprocessing

Preprocessing software	N/A
Normalization	N/A
Normalization template	N/A
Noise and artifact removal	N/A
Volume censoring	N/A

Statistical modeling & inference

Model type and settings	N/A

Effect(s) tested	N/A

Specify type of analysis: ☐ Whole brain ☐ ROI-based ☐ Both

Statistic type for inference (See Eklund et al. 2016)	N/A
Correction	N/A

Models & analysis

n/a	Involved in the study
☒	☐ Functional and/or effective connectivity
☒	☐ Graph analysis
☒	☐ Multivariate modeling or predictive analysis
